# Homœopathic Dilutions

**Published:** 1892-02

**Authors:** Rufus Choate

**Affiliations:** Washington, D. C.


### HOMŒOPATHIC DILUTIONS.

RUFUS CHOATE, M. D., WASHINGTON, D. C.

The editorial in THE HOMŒOPATHIC PHYSICIAN for October, 1891, is, I think, open to criticism. In the illustration of the marbles in the cigar box, suppose the bottom of that box is perforated, and your desire is to send the curative marble through the box into the field where the cause of the disease exists. Taking out a certain number of marbles will not diminish the size of the last marble to a stage capable of passing through the perforated bottom.

Whilst I have spoken only of the *bottom* of the box being perforated, actually to make a clearer representation of the subject, the top also of the box should be perforated with holes very much smaller than the diameter of the marble within the box. Now, when the attrition of the marbles by shaking and their loss of weight by friction have become sufficient to pass out through these small holes they will be the representative of the potentized drug.

Permit me to refer to the manuscript of my ten-years-hence-to-be-published book and quote the following:

" Minuteness of dose does not mean the spiritualization of the drug. Diseases are not entities, to be sure, but when they reach the comprehension of man they are material efforts appearing upon a material plane. Potentiation, or, if you prefer the word attenuation, spiritualizes nothing. He who can measure, analyze, and thoroughly describe the shape of an atom of the first potency will as surely believe in its materiality as he will believe in the materiality of the crude drug. If the first potency does not remove the drug from the material plane neither will the 100,000. That an atom passes out of the range of our present ken does not prove its loss of materiality; greater powers to-morrow will make it distinctly visible and show it the prey of thousands of foes smaller than itself. We may potentiate, or attenuate to the end of the finite, and the material will be none the less matter," etc.

Let us return more especially to the question, Why is it that common salt can be taken as food and not act at the same time curatively where Chloride of Sodium is the appropriate drug?

Suppose the body to be divided into one hundred sections and the meshes of each section to be one thousand times finer than the meshes of the preceding lower section. Number these sections from No. 1, the lowest and most external to No. 100, the highest and most interior. Suppose again that the disease for which Natrum-mur. is the appropriate remedy has penetrated the system to the depth of the fiftieth section. The crude common salt will enter only so far as its atoms can penetrate through the meshes; say at section No. 1. Then potentiation, that grand discovery of our honored master alone, can avail to carry the benign influence of common salt to the portion of the system where it is needed to cure the disease.

